# Brazilian adaptation of the Addenbrooke’s Cognitive Examination-Revised (ACE-R)

**DOI:** 10.1590/s1980-57642008dn10200015

**Published:** 2007

**Authors:** Viviane Amaral Carvalho, Paulo Caramelli

**Affiliations:** 1 Post-graduate program in Neurology, University of Sao Paulo School of Medicine, São Paulo (SP), Brazil.; 2 Behavioral and Cognitive Neurology Unit, Department of Internal Medicine, Faculty of Medicine of the Federal University of Minas Gerais, Belo Horizonte (MG), Brazil.

**Keywords:** dementia, diagnosis, cognitive evaluation, Addenbrooke’s Cognitive Examination-Revised, cultural adaptation, Brazil

## Abstract

The Addenbrooke’s Cognitive Examination-Revised (ACE-R) is a highly sensitive and specific tool for the detection of mild dementia. It is particularly useful in differentiating Alzheimer’s disease from frontotemporal dementia. While the first version of the test battery has been adapted in many countries, its revised version has not, probably because it was published very recently.

**Objective:**

To translate and adapt the ACE-R for use in the Brazilian population.

**Methods:**

Two independent translations were made from English into Portuguese, followed by two independent back-translations. Few adaptations in accordance to the Brazilian culture and language were made and a first version of the instrument produced. This former version of the ACE-R was administered to 21 cognitively healthy subjects aged 60 years or more, with different educational levels.

**Results:**

The mean age of the studied sample of healthy elderly was 75.4 years (ranging from 60 to 89 years). Small additional modifications were necessary after the evaluation of the first ten subjects in order to improve comprehension of the test. The final Portuguese version of the ACE-R was produced and was found to be well understood by the remaining 11 subjects, taking an average of 15 minutes to be administered.

**Conclusions:**

The Brazilian version of the ACE-R proved to be a promising cognitive instrument for testing both in research and clinical settings. With this regard, additional studies are currently being carried out in our unit in order to investigate the diagnostic properties of the ACE-R in our milieu.

## Abstract (pt)

A Addenbrooke’s Cognitive Examination - Versão Revisada (ACE-R) é um instrumento com elevada sensibilidade e especificidade para detectar demência em estágio leve. Ela é particularmente útil para diferenciar a doença de Alzheimer da demência frontotemporal. Enquanto a primeira versão desta bateria foi adaptada em vários países, a versão revisada ainda não, provavelmente por ter sido publicada muito recentemente.

**Objetivo:**

Traduzir e adaptar a ACE-R para uso na população brasileira.

**Métodos:**

Foram feitas duas traduções independentes do Inglês para o Português, seguidas de duas retro-traduções também independentes. Algumas adaptações de acordo com a cultura brasileira e a língua portuguesa foram realizadas e a primeira versão brasileira do instrumento produzida. Esta foi administrada a 21 sujeitos saudáveis com idades de 60 anos ou mais, com diferentes níveis de escolaridade.

**Resultados:**

A média de idade da amostra de idosos estudada foi de 75,4 anos (variando de 60 a 89 anos de idade). Após a avaliação dos dez primeiros indivíduos foi necessário realizar pequenas alterações com o objetivo de melhorar a compreensão do teste. A versão final em Português da ACE-R foi produzida e se mostrou de fácil entendimento pelos onze participantes restantes, tendo tempo médio de aplicação de 15 minutos.

**Conclusões:**

A versão brasileira da ACE-R provou ser um instrumento promissor de avaliação cognitiva promissor para a pesquisa e para a prática clínica. Estudos adicionais estão sendo realizados em nossa unidade para investigar as propriedades diagnósticas da ACE-R em nosso meio.

Cognitive evaluation is a mandatory step for the diagnosis of dementia, warranting more attention in Brazil. There are still only a limited number of instruments that present specific norms for use in our population, especially regarding the variables age and education. In Brazil, the available instruments for brief cognitive evaluation of dementia, in particular for Alzheimer’s disease (AD) include:^1^ the Mini-mental State Examination (MMSE),^2-6^ the Test of Information-Memory-Concentration (IMC) of Blessed,^7^ the CASI-S (Cognitive Abilities Screening Instrument - Short Form),^8-10^ the Consortium to Establish a Registry for Alzheimer’s Disease (CERAD),^11,12^ the Cambridge Examination for Mental Disorders of the Elderly (CAMDEX),^13,14^ the NEUROPSI^15,16^ and the Alzheimer’s Disease Assessment Scale Cognition Component (ADAS-cog).^17,18^ Another tool also used in our population is the Brief Cognitive Screening Battery (BCSB)^19-22^ that consists of the presentation of a sheet of paper with 10 simple line drawings that evaluates naming, recall and recognition aspects of memory, using a semantic verbal fluency test and the clock drawing test as interference tasks.^23^

The Addenbrooke’s Cognitive Examination (ACE) is an additional tool which has similar characteristics, although has not been studied in our population to date. The ACE is a brief and reliable test battery that provides detection of early stages of dementia and is also efficient in differentiating its subtypes, such as AD, frontotemporal dementia (FTD), progressive supranuclear palsy, and other forms of dementia associated with parkinsonism.^24^ The test can be administered in 15 to 20 minutes and, together with the MMSE, provides a more thorough evaluation of six cognitive domains (orientation, attention, memory, verbal fluency, language and visuospatial ability). Each of these domains can be individually evaluated. Moreover, Mathuranath et al.^24^ developed a ratio (V+L) / (O+M), called the *VLOM ratio*, after observing that AD patients presented better performance in domains such as verbal fluency (V) and language (L) when compared to FTD patients. These findings are similar to the neuropsychological profiles of both diseases observed in other studies.^25-28^ However, patients with FTD presented better performance in orientation tasks (O) and episodic memory (M), compared to AD patients.

The Addenbrooke’s Cognitive Examination was subsequently revised and data on this new version has recently been published by Mioshi et al.^25^ In this new version, called ACE-R, the structure and the sequence of the tasks were completely reworked in order to facilitate its use. The content was also modified to facilitate future translations, allowing adaptations and use in other cultures, besides slightly increasing the instrument’s sensitivity level. Furthermore, the ACE-R now has three versions (A, B and C). These versions differ between each other with regard to the anterograde memory task, in which three different stimuli are offered for the item “name and address”, in order to prevent recalling information from previous assessments. Another difference from the original version was that instead of six, the ACE-R assesses only five domains, namely: orientation and attention (18 points), memory (26 points), verbal fluency (14 points), language (26 points) and visuospatial ability (16 points). The individual’s total score is still obtained by the addition of all subtests’ scores, ranging from 0 to 100.

The authors also developed a guide of instructions to offer further information on how the scores should be noted. This guide also presents examples of acceptable answers, models of drawings, and explanations of how to apply and correct the test. The idea is to create a pattern when organizing all the answers in order to improve the overall reliability of the test among examiners.

While the original version has previously been studied in several countries, such as Germany,^29^ Argentina,^30^ Belgium,^31,32^ Spain,^33^ India^34^ and Israel,^35^ the ACE-R has not yet been studied outside the UK. The present article has the objective of presenting the adapted version of the ACE-R for use in Brazil.

## Methods

### Methodology of adaptation / Portuguese version of ACE-R

The process of ACE-R adaptation was initiated by two independent translations from English to Portuguese, followed by two back-translations of these Portuguese versions into English. The aim of this work was to reveal the possible misunderstandings and ambiguities that the first translated version could have contained. Subsequently, we carried out cross-cultural adaptation. The same method was used for the Portuguese version of the Instructions guide.

In this adaptation of the battery we used the Brazilian version of the MMSE proposed by Brucki et al.,^14^ as recommended by the Department of Cognitive Neurology and Aging of the Brazilian Academy of Neurology.^1^ The name and address pertaining to the memory item were modified, as well as the questions of the retrograde memory item (the current president’s name and the name of the president who proposed and built the federal capital Brasília, in the early 50s). In the Language-naming item, the illustration of a pencil was substituted by a pen, in order to maintain the same command as the MMSE version mentioned earlier. In the Language-comprehension item, one of the four commands was also substituted and the illustration of an alligator inserted, so that the individual could “point to the figure found at the Pantanal”, a swamp area and a well known Brazilian ecosystem. In the Language-reading, irregular words were chosen to have similar levels of difficulty in Portuguese and in English, such as “táxi, testa, saxofone, fixar and ballet” (cab, forehead, saxophone, to fix and ballet).

The ACE-R Brazilian version instrument took its final form after a pilot study test. In this study, 21 healthy individuals were tested, aged 60 years or more, with different educational levels, but no illiterate subjects were included. All participants had no history of neurologic or psychiatric disease, as well as no history of cognitive decline. They were all fully independent and performed above specific education-adjusted cut-off scores in the MMSE as suggested by Brucki et al.,^6^ namely: ≥22 for subjects with 1-4 years, ≥25 for 5-8 years and ≥26 for those with 9 or more years of schooling. Initially, ten subjects were submitted to the Portuguese first version and this experience highlighted some additional modifications which were necessary. Following this, the final Portuguese version of the ACE-R was produced and then administered to the remaining 11 participants.

The study was approved by the Ethics Committee of the Hospital das Clínicas of the University of São Paulo School of Medicine and by the Ethics Committee of the Federal University of Minas Gerais. All participants signed the written informed consent.

## Results

The 21 elderly subjects presented a mean age of 75.4± 7.1 years, ranging from 60 to 89 years. Seventeen (80.9%) of the participants were female and four (19.1%), male. The mean number of years of formal education was 8.5±4.3(ranging from 3 to 22 years).

The lowest total score observed in the ACE-R was 73, while the highest was 98. The mean total score in the battery was of 83.3±10.0points. Minimum and maximum total scores observed, as well as means and standard deviations for the subtests of the battery, are shown in Table 1. The instrument took 15 minutes on average to be administered. Overall, the comprehension of the different items of the test was found to be good.

**Table 1:** Main demographic data of the sample and ACE-R scores

N=21	Minimum	Maximum	Mean (SD)	Maximum score
Age	60	89	75.4 (7.1)	-
Schooling *(in years)*	3	22	8.5 (4.3)	-
MMSE	22	29	26.9 (2.2)	30
**ACE-R scores**				
Attention and orientation	13	18	16.5 (1.5)	18
Memory	14	25	20.0 (4.0)	26
Fluency	4	13	10.1 (2.2)	14
Language	14	26	22.9 (3.4)	26
Visuospatial	9	16	14.2 (1.8)	16
Total score	73	98	83.3 (10.0)	100

## Discussion

In this study we have translated and adapted the revised version of the ACE (ACE-R) to be used in Brazil. After its application on half of the sample, some additional modifications were made to make it easier to understand by the low education level subjects. Obviously, these modifications did not interfere with the original aims of the battery authors. The final instrument proved to be easy to administer and was well understood by a group of healthy elderly people with heterogeneous educational background.

Cognitive evaluation constitutes an important tool for the assessment of cerebral functioning, being mandatory for the differential diagnosis between normal aging, mild cognitive impairment, dementia and its subtypes. Sensitivity and specificity are essential aspects of a cognitive investigative instrument, but these also depend on knowledge of the imperative diversities and influences between cultures, gender, age and educational level.

As outlined previously, brief cognitive evaluation instruments validated in Brazil remain scarce. Moreover, the differential diagnosis between AD and FTD represents a challenge, especially in the early stages, and even fewer tools are available in our milieu with this regard. Recently, a Brazilian version of the Frontal Assessment Battery (FAB) was published by Beato et al.^36^ This study represents an important contribution for clinicians since it describes the performance of a group of healthy elderly people in an executive function brief evaluation tool. Previous studies have shown good sensitivity of the FAB in detecting frontal lobe dysfunction^37^ and also in differentiating AD from FTD patients.^38-40^

However, the FAB assesses only some aspects of executive functions, while the ACE-R includes the assessment of different cognitive domains, such as orientation and attention, memory, verbal fluency, language and visuospatial ability. The more comprehensive evaluation together with the VLOM ratio analysis provided by the ACE and also by the ACE-R offer more information on the patients’ neuropsychological profiles, which can be helpful to identify specific dementia subtypes. In the original version (ACE), patients with AD performed better on tests of verbal fluency and language (VL), whereas patients with FTD presented relatively better results on orientation and episodic memory tasks (OM).^24,30,33^ Similar findings were reported using the revised version of ACE.^25^ Therefore, the Brazilian version of the ACE-R seems to be a promising tool for clinical use and therefore warrants validation in our population. The Brazilian version of the ACE-R is available through contact with the authors.

These are the preliminary results of a study that intends to investigate the applicability of ACE-R as an instrument of brief cognitive evaluation for healthy Brazilian subjects and patients with suspected dementia, to determine the sensitivity and specificity of this tool in mild-AD patients and to later assess its capacity to differentiate between AD and FTD patients.
